# Health behaviours and telomere biology in severe mental disorders

**DOI:** 10.1038/s41398-026-04362-2

**Published:** 2026-09-02

**Authors:** Vid Mlakar, Marta Di Forti, Els F. Halff, Deepak P. Srivastava, Ibrahim Akkouh, Srdjan Djurovic, Carmen Martin-Ruiz, Daniel S. Quintana, Viktoria Birkenæs, Nils Eiel Steen, Monica B. E. G. Ormerod, Ole A. Andreassen, Monica Aas

**Affiliations:** 1 Social, Genetic and Developmental Psychiatry Centre, Institute of Psychiatry, Psychology and Neuroscience, King’s College London, London, UK; 2 South London and Maudsley NHS Foundation Mental Health Trust, London, UK; 3 Department of Basic and Clinical Neuroscience, Institute of Psychiatry, Psychology and Neuroscience, King’s College London, London, UK; 4 MRC Centre for Neurodevelopmental Disorders, King’s College London, London, UK; 5 Department of Medical Genetics, Oslo University Hospital, Oslo, Norway; 6 Centre for Precision Psychiatry, Division of Mental Health and Addiction, University of Oslo, Oslo, Norway; 7 BioScreening Core Facility-CAV, Ageing Research Laboratories, Campus for Ageing and Vitality, Newcastle University, Newcastle, UK; 8 Department of Psychology, University of Oslo, Oslo, Norway; 9 NevSom, Department of Rare Disorders, Oslo University Hospital, Oslo, Norway; 10 Section for Clinical Psychosis Research, Division of Mental Health and Addiction, Oslo University Hospital, Oslo, Norway; 11 Department of Psychiatric Research, Diakonhjemmet Hospital, Oslo, Norway; 12 Department of Psychosis Studies, Institute of Psychiatry, Psychology and Neuroscience, King’s College London, London, UK

**Keywords:** Schizophrenia, Predictive markers, Bipolar disorder

## Abstract

Alterations in telomere length (TL), a marker of cellular ageing, have been reported in individuals with severe mental disorders (SMD). Epidemiological studies of the general population have highlighted that unhealthy lifestyles may exacerbate telomere attrition. However, the impact of lifestyle on TL within the context of SMD remains unexplored. The study consisted of 410 participants (schizophrenia spectrum [*n* = 225] and affective disorder [*n* = 185]) collected as part of the Norwegian Thematically Organised Psychosis (TOP) study. Leukocyte TL was measured via blood and determined by quantitative real-time Polymerase Chain Reaction (qPCR). Patients provided self-report data on six lifestyle domains including: diet, exercise, smoking, alcohol consumption, substance use, and coffee consumption. A global dichotomised (healthy vs unhealthy) lifestyle variable was created, as well as a health behaviour variable, indicating the level of unhealthy behaviours. Individuals with an unhealthy lifestyle had shorter TL compared to those with a healthy lifestyle (Cohen’s d = 0.58, F = 8.62, *p* = 0.004). A relationship was observed between increasing number of unhealthy behaviours and shorter TL (F = 2.69, *p* = 0.02), adjusted for age, sex, ethnicity, trauma exposure, medication daily defined dose (DDD), years of education and diagnosis. In terms of base-pair loss, individuals with healthy lifestyles exhibited a roughly 6-year lower biological age, compared to individuals with unhealthy lifestyles. Our study indicates that a healthier lifestyle is associated with longer TL in SMD. This highlights the importance of health behaviours as potential clinical targets for ensuring healthier cellular ageing in psychiatric populations.

## Introduction

Individuals affected by severe mental disorders (SMD), such as bipolar and psychotic disorders, have a significantly shorter lifespan when compared to unaffected peers, equal to approximately 15–20 years [1]. In addition to mortality rate, individuals with SMD tend to be disproportionately affected by somatic diseases associated with advanced chronological age, such as metabolic syndromes, cardiovascular disease, and dementia [2, 3]. One proposed explanation for these differences is that individuals with SMD may possess a faster rate of biological ageing [4], as assessed through measurement of telomere length (TL).

Telomeres are non-coding endpoints of human chromosomes, comprised of repeating TTAGGG nucleotides [5]. The main function of telomeres is to protect chromosomal ends during DNA replication [6]. Telomeres shorten both through successive cell divisions, and due to cellular and environmental stressors. Telomere length can be extended by telomerase; however, this enzyme is inactive in most somatic cells in the human body [7]. Once a critical length is reached, cells will enter a state of replicative senescence or undergo apoptosis [8], slowing tissue and organ renewal [9]. This is thought to be one of the main processes underlying ageing [10].

Telomere attrition affects all humans; however, epidemiological studies of TL suggest differences between the sexes [11], across ethnicities [12], and more recently, among individuals with SMD [4]. While variability exists, studies in psychiatric populations have noted that individuals with SMD tend to possess shorter telomeres compared to healthy controls [13, 14]. Similar variability is found when examining the expression of genes responsible for telomere maintenance, telomerase reverse transcriptase (*TERT)* and telomerase RNA component (*TERC)* [15], which comprise the key telomere maintenance enzyme, telomerase [16]. One mechanism which has been proposed is that individuals with SMD have less healthy lifestyle behaviours compared to unaffected controls [17].

Reviews of SMD patient lifestyles have consistently noted an increased number of unhealthy behaviours such as poor diet, low physical activity, smoking and substance use [17]. This has been argued to be partly a result of symptom interference [18] and medication side effects, such as lethargy and increased appetite [19, 20]. These are often compounded by adverse childhood experiences [21], which are prevalent in SMD. Analyses of these health domains have supported their influence on TL shortening. Particularly, substance use has been found to significantly reduce TL [22] across a wide range of substances. This may be due to the increase in oxidative stress and inflammation caused by such substances [23, 24]. In addition, evidence from the general population indicates reduced TL in individuals reporting poorer diet [25], sedentary behaviour [26], and smoking [27]. These are all behaviours associated with oxidative stress and inflammation [28, 29], to which TL is sensitive [30]. Interestingly, coffee consumption seems to be associated with better telomere maintenance [31]. This has been speculated to potentially be the result of the antioxidant properties of coffee [32].

Similarly, evidence indicates that telomere maintenance mechanisms are impacted by lifestyle. Most notably, research into exercise has indicated upregulated *TERT* expression in both individuals engaging in casual exercise, as well as endurance athletes [33, 34]. Moreover, research into substance use has noted reductions in telomerase activity in individuals using heroin [35] and downregulation of *TERT* expression with exposure to cannabis [36]. In contrast, there is a lack of literature examining the expression of *TERC* in relation to lifestyle.

However, despite the apparent association between individual health factors and TL, few studies examine the association between TL and global lifestyle across several different domains, including diet, exercise, and substance use. One study [37] found longer telomeres in middle-aged women who maintained healthier lives at one-year follow-up. Similarly, only one study has examined telomerase activity in relation to lifestyle at 5-year follow-up, indicating little association between overall lifestyle and telomerase [38]. Yet, despite their findings, a notable gap in the literature exists due to such studies being carried out in the general population. Thus, it is difficult to generalise these findings to the SMD population, where biological ageing might already be accelerated.

The main aim of the current study is to provide a comprehensive evaluation of how health behaviours may interact with TL in individuals with SMD. To the best of our knowledge, there are no previous studies investigating the cumulative effect of lifestyle/health behaviours on TL in this specific population. Based on previous literature, we anticipate that 1) TL will decrease with increasing unhealthy behaviours, 2) individuals with the healthiest lifestyles will possess the longest telomeres.

## Methods

### Participants

The study encompassed 410 participants with SMD (schizophrenia spectrum [*n* = 225] and affective disorders [*n* = 185]), selected from the Norwegian Thematically Organised Psychosis (TOP) study, who were recruited between 2007 and 2018 [39]. Participants were recruited from four psychiatric units across Oslo, Norway. Participants were excluded based on the following criteria: age outside the 18–65 range, not fluent in a Scandinavian language, or having a current or past diagnosis of organic psychosis [39]. All participants provided informed consent, with the study being approved by both the Regional Committee for Medical Research Ethics and the Norwegian Data Inspectorate (2009/2485/REK Sør-Øst).

### Health behaviours

The health behaviours analysed in the study comprised diet, exercise, alcohol consumption, smoking, substance use, and coffee consumption. To determine alcohol consumption the Alcohol Use Disorder Identification Test (AUDIT) [40] was used. Following scoring guidelines, sum scores ranging from 1–7 indicate low-risk alcohol consumption (‘healthy’ group), and scores above 8 indicate harmful alcohol use (‘unhealthy’ group).

Self-report on illicit substance use was provided by participants and dichotomised into those with any lifetime substance use and those without any substance use. The substances included within the assessment were: cannabis, cocaine, heroin, hallucinogens, amphetamines, opioids, and PCP. Information on daily smoking was provided by participants and dichotomised into daily smoking and non-smoking groups.

Self-report information regarding exercise duration was provided by participants and split into ‘unhealthy’ (less than 150 min per week) and ‘healthy’ (more than 150 min per week) groups based on the World Health Organisation (WHO) recommendation of a minimum weekly exercise duration of at least 150 min [41].

Assessment of diet was based on interviews with patients of diet patterns during the last six months and grouped into healthy to poor diet following WHO guidelines of healthy diet [42]. Due to previous findings associating coffee consumption with longer telomeres in the sample [43], we included information on coffee consumption. The data was provided as a self-report variable, which was dichotomised into coffee drinkers (healthy) and non-drinkers (unhealthy).

Subsequently, participants were scored based on the number of unhealthy behaviours they exhibited (min score 0 and max score 6), to produce a lifestyle variable. The results were dichotomised into a ‘healthy’ (participants reporting 0 unhealthy behaviours; *n* = 37) vs ‘unhealthy’ (participants reporting 1 or more unhealthy behaviours; *n* = 373) lifestyle. In addition, a health behaviour variable ranging from 0 to 5 was also formed. The latter was done as only one participant (from the affective disorders group) possessed 6 unhealthy behaviours. In effect, groups 6 and 5 were collapsed into group 5. For an overview of N for each respective group, please see Supplementary Material Table S1.

In addition to health behaviours, the study also included data on childhood trauma exposure, evaluated using the childhood trauma questionnaire (CTQ) [44]. For more information about trauma operationalisation, see Aas et al. [45]. Lastly, patient records were used to calculate the daily defined dose (DDD) of individual psychotropic medication (antipsychotics, antidepressants, lithium, and mood stabilisers).

### Telomere length

Telomere length was assessed in all participants using peripheral blood leukocytes, through a quantitative real-time polymerase chain reaction (qPCR) method [45, 46]. The qPCR involved the extraction of 10 ng of DNA from leukocyte cells, which was combined with 5 μl of SYBR®Green JumpStart Taq Ready Mix and 0.25 μl of ROX reference dye. All assessments were carried out using a 384-well plate Applied Bio- systems 7900HT Fast Real Time qPCR. All inconclusive or extreme samples (top and bottom 5%) were re-evaluated. For further details on the methods, see Mlakar et al. [46].

The qPCR provided researchers with a telomere to single copy ratio (T/S ratio), which was used to estimate mean TL. Smaller T/S ratios indicated shorter mean TL. Analyses of variation revealed that the sample possessed an intra-assay coefficient of 6.07% and an inter-assay coefficient of 6.08%. All blood samples were stored in The Biobank, located in Oslo, Norway.

In addition to the T/S ratio provided by the qPCR analysis, we estimated differences in base-pair attrition. This was carried out using a previously proposed quantitative estimate of years of accelerated aging, with an average of 70 base-pair reduction per year [47]. To estimate TL attrition, we subtracted the base-pair differences between different groups (e.g.: mean TL in healthy lifestyle - mean TL in unhealthy lifestyle) and divided the difference by 70. The end result provides a rough estimate of TL attrition.

### TERT & TERC expression

The expression of *TERT* and *TERC* was quantified using microarrays, following methodology previously detailed in Akkouh et al. [48]. Blood samples were collected from participants in Tempus Blood RNA Tubes, with total RNA being extracted using the TEMPUS 12-Port RNA Isolation Kit (Thermo Fisher). The quantification of genetic expression itself was performed using the Illumina HumanHT-12 v4 Expression BeadChip (Illumina). Multidimensional scaling and hierarchical clustering were employed as part of standard quality control (sample quality measurements and removal of outliers) as well as for the purposes of removing batch effects (RNA extraction batch, RNA extraction method, DNase treatment batch, cRNA labelling batch, and chip hybridization). Overall batch-adjusted and log2-transformed values of *TERT* and *TERC* expression were used for statistical analysis.

### Statistical analysis

All statistical analyses were performed using IBM SPSS v26 software. For categorical variables chi-squared tests were used to compare the distribution between the two groups (patients with schizophrenia and affective disorders). Due to the potential influence of childhood trauma on both lifestyle and TL [21, 45], all analyses were adjusted for this factor. Additionally, several environmental confounders were tested in regard to their association with either TL or lifestyle, including BMI, employment, and years of education. Only years of education were significantly associated with lifestyle, leading to their inclusion as a covariate. To investigate associations between TL and lifestyle we performed an ANCOVA, comparing TL between the healthy lifestyle group (0 unhealthy behaviours) and the unhealthy lifestyle group (1 or more unhealthy behaviours). Additionally, used an ANCOVA to perform a sensitivity analysis, comparing an amended healthy lifestyle group (up to 1 unhealthy behaviour) to the unhealthy lifestyle group (2 or more unhealthy behaviours). Finally, we used an ANCOVA to compare TL between unhealthy behaviours, ranging from 0 to 5 (see Supplementary Material Table S1 for an overview of N in the different lifestyle groups). All analyses were adjusted for age, sex, ethnicity, childhood trauma, daily defined dose (DDD) of psychotropic medication, years of education, and diagnosis, with Bonferroni post-hoc corrections. Cohen’s d effect size was also calculated. Due to skewness, TL were log-transformed before being added to the parametric models.

## Results

### Demographic overview

Table 1 provides the descriptive statistics for the study sample (*n* = 410). In summary, the schizophrenia group was significantly younger and had a higher proportion of men than the affective disorder group. There was no significant difference in ethnicity between the diagnostic groups, with both consisting primarily of individuals with European ancestry.

**Table 1:** Demographic Overview.

Variable	SZ (*n *= 225)	AD (*n *= 185)	Statistics	Post hoc analyses
Age (years), mean (SD)	28.57 (9.39)	32.10 (11.99)	*F *= 11.18, *p *< 0.001	SZ < AD
Sex				
Male, N (%)	132 (58.7)	83 (44.9)	*χ*^*2*^ = 7.75, *p* = 0.005	SZ > AD
Ethnicity				
European, N (%)	184 (81.8)	156 (84.3)	*χ*^*2*^ = 0.46, *p* = 0.5	-
Years of education, mean (SD)	12.99 (2.81)	14.29 (3.02)	*F* = 18.62, *p* > 0.001	SZ < AD
Telomere Length (T/S ratio), mean (SD)	1.12 (0.26)	1.16 (0.27)	*F* = 2.97, *p* = 0.09	-
Lifestyle				
Unhealthy, N (%)	208 (92.4)	165 (89)	*χ*^*2*^ = 1.31, *p* = 0.25	-
Coffee Consumption				
No, N (%)	29 (12.9)	31 (16.8)	*χ*^*2*^ = 1.216, *p* = 0.3	-
Healthy Diet				
No, N (%)	83 (36.9)	57 (30.8)	*χ*^*2*^ = 1.668, *p* = 0.2	-
Healthy Exercise				
No, N (%)	119 (52.9)	83 (44.9)	*χ*^*2*^ = 2.615, *p* = 0.1	-
Daily Smoking				
Yes, N (%)	138 (61.3)	101 (54.6)	*χ*^*2*^ = 1.896, *p* = 0.2	-
Substance Use				
Yes, N (%)	59 (26.2)	51 (27.6)	*χ*^*2*^ = 0.094, *p* = 0.7	-
Risky Alcohol Consumption				
Yes, N (%)	83 (36.9)	73 (39.5)	*χ*^*2*^ = 0.285, *p* = 0.6	-
Trauma exposure, mean (SD)	44.53 (15.39)	42.63 (15.85)	*F* = 1.39, *p* = 0.24	-
Antipsychotic Dose (mg/day), mean (SD)	1.58 (4.06)	0.68 (0.89)	*F* = 7.95, *p* = 0.005	SZ > AD
Antidepressant Dose (mg/day), mean (SD)	0.44 (0.87)	0.47 (0.99)	*F* = 0.10, *p* = 0.75	-
Mood Stabiliser Dose (mg/day), mean (SD)	0.08 (0.26)	0.49 (0.64)	*F* = 72.88, *p *< 0.001	SZ < AD
Lithium Dose (mg/day), mean (SD)	0.01 (0.11)	0.24 (0.51)	*F* = 37.68, *p *< 0.001	SZ < AD

*SZ* schizophrenia spectrum, *AD* affective disorders, *n* number, *SD* standard deviation, *mg/day* milligrams per day, *T/S ratio* telomere to single copy gene ratio. (For telomere length, statistics [F and *p*-value] are based on log transformation and adjusted for age, sex and ethnicity).

With respect to medication, data was available for 379 participants. Of these, in the schizophrenia sample, 83.1% were taking antipsychotic medication, 2.3% lithium, 26.8% antidepressant medication, and 13.6% other mood stabilisers. In the affective disorder group, 59% were taking antipsychotic medication, 22.6% lithium, 28.9% antidepressant medication, and 52.4% other mood stabilisers. The affective disorder group showed a trend towards longer telomeres (F = 2.97, *p* = 0.09), as well as a significantly longer time spent in education (*F* = 18.62, *p* > 0.001) compared to the schizophrenia group. There was no statistical difference concerning childhood trauma exposure between groups (*p* > 0.1).

### Lifestyles in severe mental disorders

Overall, there were no significant differences between the two diagnostic groups for any of the individual health behaviour domains (exercise, diet, smoking, coffee consumption, alcohol consumption, substance use, all *p* > 0.1, Table 1). Equally, there were no significant differences in global lifestyle between the two diagnostic groups (*χ*^*2*^ = 1.31, *p* = 0.25). For an overview of the association between the health behaviours, please see Supplementary Material Table S2.

In terms of the association between lifestyle and trauma, sexual abuse was significantly associated with a globally unhealthy lifestyle (F = 4.29, *p* = 0.04, Figure S1), as well as increasing unhealthy behaviours (F = 3.15, *p* = 0.008, Figure S2), adjusted for age, sex and ethnicity. None of the other trauma subtypes (physical abuse, emotional abuse, physical neglect, and emotional neglect) were associated with lifestyle (all *p* > 0.5). These results remained after Bonferroni corrections for multiple testing.

### Lifestyle and telomere biology

**Fig. 1:**
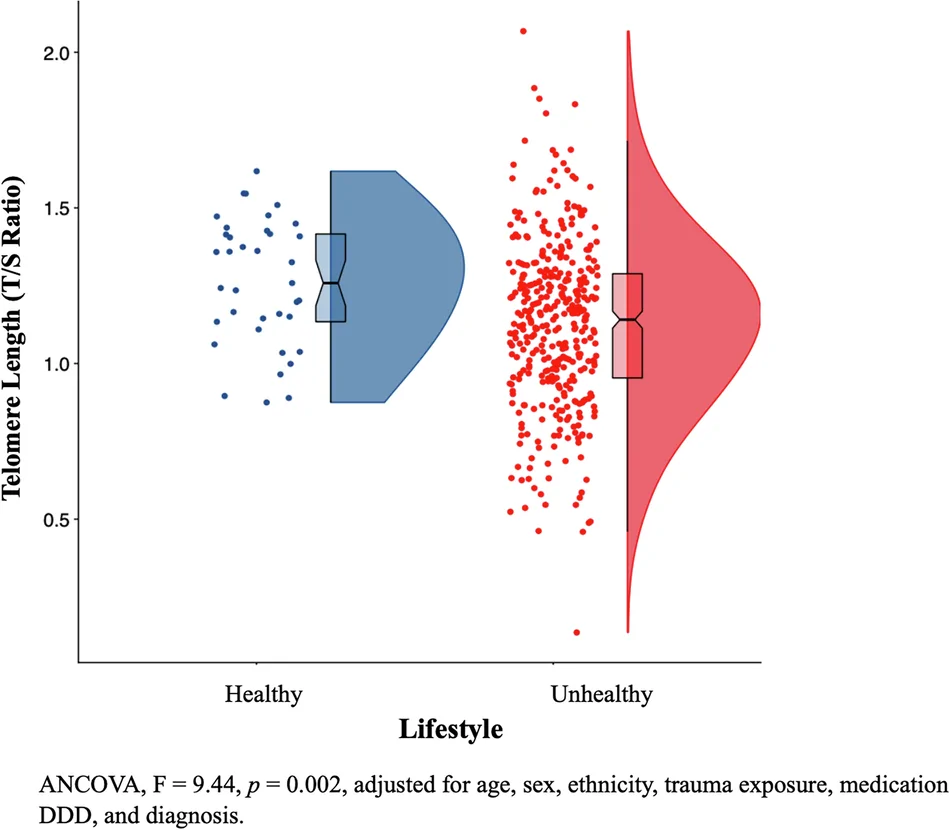
Patients with an unhealthy lifestyle have shorter telomeres than patients with a healthy lifestyle.

**Fig. 2:**
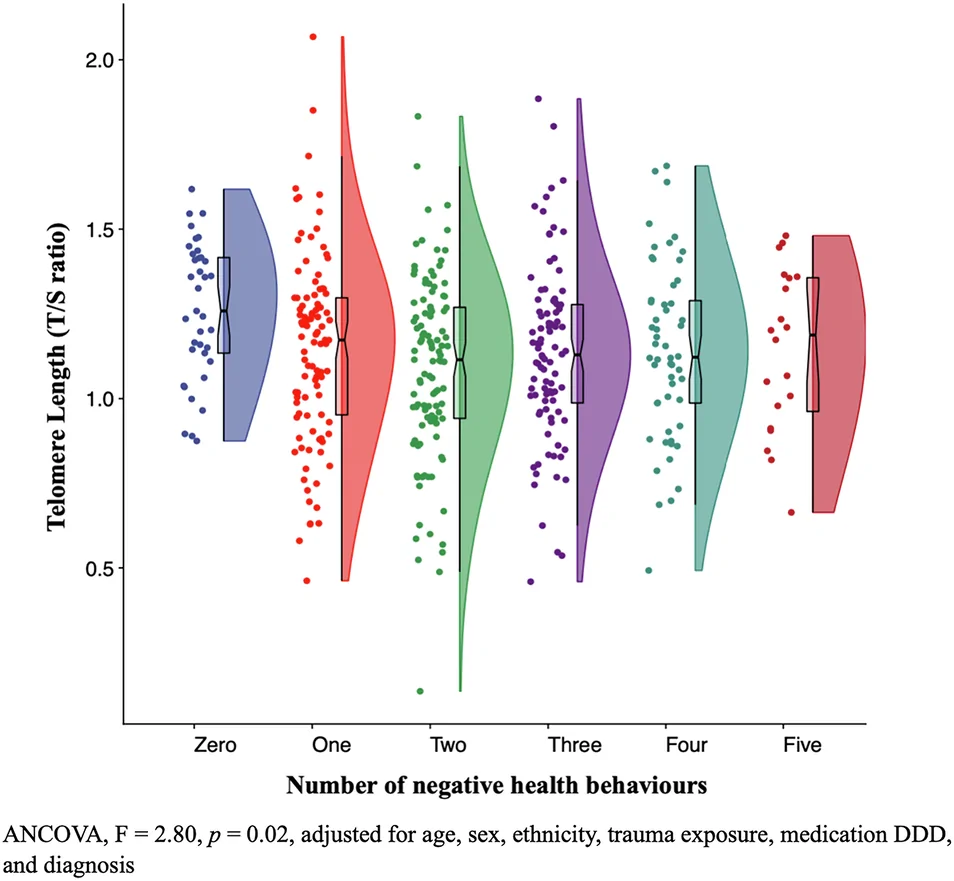
Increasing number of unhealthy behaviours is associated with shorter telomeres.

Patients with an unhealthy lifestyle had shorter TL than patients with a healthy lifestyle (Cohen’s d = 0.58, F = 8.62, *p* = 0.004, see Fig. 1). Sensitivity analyses were performed, including up to 1 unhealthy behaviour in the healthy lifestyle group, with the association remaining significant (F = 5.69, *p* = 0.02, see Supplementary Material Figure S3). For a complete overview of the number of negative health behaviours and TL (F = 2.69, *p* = 0.02), please see Fig. 2. All TL analyses described above were adjusted for age, sex, ethnicity, trauma exposure, diagnosis, years of education, and medication.

In terms of base-pairs, individuals with a healthy lifestyle possessed on average 5293.37 (+/−791.39) base-pairs, whereas individuals with unhealthy lifestyles had an average of 4857.62 (+/−1092.98) base-pairs. This difference amounts to a roughly 6-year lower biological age of the healthy group (F = 6.21, *p* = 0.02), adjusted for age, sex, ethnicity, trauma exposure, diagnosis, years of education, and medication.

There was no significant association between *TERT* expression and either dichotomous lifestyle (F = 0.41, *p* = 0.52) or increasing unhealthy behaviours (F = 0.70, *p* = 0.62), adjusted for age, sex, ethnicity, trauma exposure, diagnosis, years of education, and medication (see Table S3). Similarly, there was no significant association between *TERC* expression and either dichotomous lifestyle (F = 2.15, *p* = 0.14) or increasing unhealthy behaviours (F = 0.60, *p* = 0.69), adjusted for the same covariates as above.

Due to previous results indicating a protective effect of coffee consumption on TL [36], a sensitivity analysis was run using a secondary model of lifestyle, excluding coffee consumption. When run using the dichotomous lifestyle variable, the results of this new model remained significant (Cohen’s d = 0.35, F = 4.44, *p* = 0.04), adjusting for the same covariates as described above, and coffee consumption.

## Discussion

In this study of individuals with SMDs, increasingly unhealthy behaviours were associated with shorter telomeres. Individuals who reported having an unhealthy lifestyle had significantly shorter telomeres compared to those with healthy lifestyles, equating to a biological age difference of roughly six years. There was no association between any of the lifestyle measures, and the expression of telomere maintenance genes *TERT* and *TERC*.

These results align with previous research proposing a protective effect of healthy lifestyle on TL in the general population. In the study by Puterman and colleagues [37], it was highlighted that a healthier lifestyle allowed for better stress management and telomere maintenance at a one-year follow-up, in a sample of women from the general population. Similarly, assessments of individual lifestyle domains (e.g.: diet, exercise, and substance use), have all noted their impact on telomere attrition [23, 25, 26]. Moreover, our findings in relation to telomerase gene expression are supported by previous research. The study by Ornish and colleagues [38], indicated that there was no difference in telomerase activity between men enrolled in a comprehensive lifestyle programme, and those not enrolled, at a 5-year follow-up. This aligns with our findings that there was no association between *TERT* or *TERC* expression and either lifestyle measure.

Overall, our study is, to our knowledge, the first to investigate lifestyle and its association with TL, and its maintenance mechanisms, in a psychiatric sample. In effect, our findings help extend existing knowledge by highlighting the importance of promoting healthy behaviours, in a population where accelerated biological ageing may already be occurring.

An unhealthy lifestyle may influence TL through several biological pathways, such as oxidative stress and inflammation. As noted previously, several behaviours, such as smoking [49], sedentary lifestyle [50], diets low in nutrients and high in sugars [50], substance and alcohol use [23], have been associated with increases in reactive oxygen species (ROS), which have been noted to be capable of directly damaging telomeres [51]. This may be exacerbated through the upregulation of pro-inflammatory cytokines such as IL-6 and TNF-α, which themselves release further ROS [52]. It could be argued that due to sustained telomeric strain generated by oxidative stress and inflammation, cells may undergo replication and proliferation to counteract cell and tissue damage, which could lead to further telomeric shortening and senescence [10]. Although we have found limited evidence of an association between immune activation and TL in SMD in a recent cross-sectional study [53], the relationship between immune activation and TL needs to be further investigated in longitudinal studies. Additionally, while we found no significant association between TERT or TERC expression and lifestyle, it could be suggested that this reflects a compensatory mechanism. In their study, Ornish and colleagues argue that telomerase may have been upregulated in the ‘healthy’ lifestyle group only in a short-term compensatory manner, reducing after having sufficiently lengthened TL [38]. Similarly, the lack of association between lifestyle and *TERT* or *TERC* expression yet longer TL, in our study, may reflect a temporal upregulation of their transcription.

Furthermore, when investigating the impact of childhood trauma, our results indicated that only sexual abuse was significantly associated with unhealthy lifestyle behaviours, suggesting a possibly distinct role of trauma subtype in shaping health behaviour. Prior studies examining the impact of sexual abuse have noted a poorer lifestyle compared to non-exposed peers [54]. Some of the documented consequences of sexual abuse in survivors have been poor self-image, emotional dysregulation, feelings of lack of control, hypervigilance, etc. [55] It could be argued that these experiences could shape later health behaviours and lifestyle. As an example, overeating may serve as a self-soothing mechanism [56], with restrictive eating providing survivors with a sense of control over their life [57]. Overall, our findings highlight the importance of trauma-informed lifestyle interventions for survivors of childhood sexual abuse.

### Study limitations

Although not uncommon in lifestyle assessments, most of the health behaviours assessed in the study relied on self-reports by the participants. Due to the nature of the information being provided (i.e. health), the participants could potentially have misrepresented or underreported their own habits, which would require results to be interpreted with caution.

Additionally, it is important to highlight that lifestyle may be influenced by several factors outside of the remit of this study, including illness severity/symptom interference, cognitive deficits, functional impairment, or socioeconomic constraints. While our study attempted to control for possible confounders by adjusting for factors such as age, sex, ethnicity, diagnosis, years of education, and psychotropic medication use, we cannot exclude the remaining confounding of the aforementioned factors. To that end, further studies are required to explore the various factors influencing an individual’s lifestyle, in regard to biological ageing.

Moreover, whilst we have speculated that TL may have been increased due to potential antioxidant or anti-inflammatory properties of certain health behaviours, longitudinal studies are needed to investigate if peripheral antioxidant or inflammation levels influences telomere biology. Similarly, detailed information on other lifestyle behaviours, such as sleep, were lacking. Further prospective studies are needed to fully explore possible pathways through which lifestyle may influence TL.

In addition, the study is only comprised of a psychiatric sample (schizophrenia & affective disorders) without a healthy control comparison group, as well as carried out cross-sectionally. This limits our ability to understand potential causality between health behaviours and changes in TL over time. Future studies would be required to establish long term determinants of telomere attrition in psychiatric populations.

Another notable limitation was that TL was measured using qPCR, which provided us with a mean TL measure, rather than the number of short telomeres within a sample [45]. The qPCR is a well validated measure for assessing TL; however, we cannot exclude the possibility that focusing on critically short TL could have given alternative information about the relationship between health behaviours and TL. Lastly, our study only included one marker of biological ageing (TL), which could not capture the entire ageing process. Ideally several markers should have been included, such as epigenetic clocks or brain age measures.

In conclusion, our study suggests that a healthier lifestyle is associated with cross-sectionally longer TL in individuals with SMD. In addition, our study highlights the distinct influence of childhood sexual abuse on adult health behaviours. This highlights the importance of health behaviours as well as addressing trauma-specific needs, as potential clinical targets for ensuring healthier cellular ageing in psychiatric populations.

## Supplementary information

Supplementary Materials

## Data Availability

All data relevant to the study are included in the article or uploaded as supplementary information.
