# Supplementary material for: Health behaviours and telomere biology in severe mental disorders
Source: Transl Psychiatry. 2026 Sep 2;16:455. doi: 10.1038/s41398-026-04362-2 (PMC13550598; doi:10.1038/s41398-026-04362-2)
Supplement: Supplementary file 1 [file 41398_2026_4362_MOESM1_ESM.docx]

**Supplementary Materials**

**Table S1.** Health behaviour distribution between diagnostic groups

| Number of Health Behaviours | Schizophrenia (*n* = 225) | Affective Disorders (*n* = 185) |
| --- | --- | --- |
| 0, N (%) | 17 (7.6) | 20 (10.8) |
| 1, N (%) | 52 (23.1) | 48 (25.9) |
| 2, N (%) | 58 (25.8) | 49 (26.5) |
| 3, N (%) | 58 (25.8) | 34 (18.4) |
| 4, N (%) | 31 (13.8) | 23 (12.4) |
| 5, N (%) | 9 (4.0) | 11 (6.1.) |

**Table S2.** Chi-square associations between health behaviours

**Significance threshold adjusted for multiple corrections*

| Behaviour 1 | Behaviour 2 | *χ^2^* | p-value |
| --- | --- | --- | --- |
| Tobacco use | Coffee Consumption | 15.68 | **< 0.001** |
| Tobacco use | Alcohol Use | 26.74 | **< 0.001** |
| Tobacco use | Substance Use | 31.63 | **< 0.001** |
| Tobacco use | Exercise | 1.56 | 0.21 |
| Tobacco use | Diet | 0.08 | 0.77 |
| Coffee Consumption | Alcohol Use | 1.93 | 0.16 |
| Coffee Consumption | Substance Use | 0.36 | 0.55 |
| Coffee Consumption | Exercise | 1.90 | 0.66 |
| Coffee Consumption | Diet | 0.02 | 0.88 |
| Alcohol Use | Substance Use | 23.56 | **< 0.001** |
| Alcohol Use | Exercise | 0.54 | 0.82 |
| Alcohol Use | Diet | 1.03 | 0.31 |
| Substance Use | Exercise | 3.85 | 0.05 |
| Substance Use | Diet | 7.23 | 0.007 |
| Exercise | Diet | 9.79 | **0.002** |

**Figure S1.** Sexual abuse and global lifestyle

ANCOVA, F = 4.29, *p* = 0.04, adjusted for age, sex, and ethnicity. Patients with more severe sexual abuse history have unhealthier lifestyles.

**Figure S2**. Sexual abuse and number of unhealthy behaviours

ANCOVA, F = 3.15, p = 0.008, adjuste d for age, sex, and ethnicity. Patients with more severe sexual abuse history have more unhealthy behaviours.

**Table S3.** Association between TERT and TERC expression, and lifestyle

*adjusted for age, sex, ethnicity, diagnosis, trauma, years of education, and medication DDD

|  | **Dichotomous Lifestyle** | | **Unhealthy Behaviour Dose** | | | | | | **Statistics** | |
| --- | --- | --- | --- | --- | --- | --- | --- | --- | --- | --- |
| **Variable** | **Healthy** | **Unhealthy** | **0**  **(N = 24)** | **1**  **(N = 58)** | **2**  **(N = 65)** | **3**  **(N = 63)** | **4**  **(N = 35)** | **5**  **(N = 13)** | **Dose** | **Dichotomous** |
| **TERT,**  **mean (SE)** | 4.44 (0.08) | 4.45 (0.07) | 4.44 (0.08) | 4.46 (0.07) | 4.44 (0.08) | 4.46 (0.08) | 4.45 (0.07) | 4.43 (0.09) | F = 0.70,  p = 0.62 | F = 0.41,  p = 0.52 |
| **TERC,**  **mean (SE)** | 4.62 (0.10) | 4.57 (0.14) | 4.62 (0.10) | 4.57 (0.18) | 4.57 (0.12) | 4.58 (0.13) | 4.57 (0.09) | 4.59 (0.14) | F = 0.60,  p = 0.69 | F = 2.15,  p = 0.14 |

**Figure S3.** Sensitivity analysis comparing TL between healthy lifestyle (0 & 1 unhealthy behaviours) and unhealthy lifestyle (2+ unhealthy behaviours)

**Telomere Length (T/S Ratio)**

**Lifestyle**

ANCOVA, F = 5.69, *p* = 0.02, adjusted for age, sex, ethnicity, trauma exposure, years of education, diagnosis, and medication (DDD)
